# Bioinformatic prospecting and phylogenetic analysis reveals 94 undescribed circular bacteriocins and key motifs

**DOI:** 10.1186/s12866-020-01772-0

**Published:** 2020-04-06

**Authors:** Ben Vezina, Bernd H. A. Rehm, Andrew T. Smith

**Affiliations:** Centre for Cell Factories and Biopolymers, Griffith Institute for Drug Discovery, Griffith University, Nathan, Australia

**Keywords:** Antimicrobial, Bioinformatics, Pathogen, Cluster analysis, Hydrophobicity, Immunity, Antibiotics, Gram positive

## Abstract

**Background:**

Circular bacteriocins are antimicrobial peptides produced by bacteria with a N and C termini ligation. They have desirable properties such as activity at low concentrations along with thermal, pH and proteolytic resistance. There are twenty experimentally confirmed circular bacteriocins as part of bacteriocin gene clusters, with transport, membrane and immunity proteins. Traditionally, novel antimicrobials are found by testing large numbers of isolates against indicator strains, with no promise of corresponding novel sequence.

**Results:**

Through bioprospecting publicly available sequence databases, we identified ninety-nine circular bacteriocins across a variety of bacteria bringing the total to 119. They were grouped into two families within class I modified bacteriocins (i and ii) and further divided into subfamilies based on similarity to experimentally confirmed circular bacteriocins. Within subfamilies, sequences overwhelmingly shared similar characteristics such as sequence length, presence of a polybasic region, conserved locations of aromatic residues, C and N termini, gene clusters similarity, translational coupling and hydrophobicity profiles. At least ninety were predicted to be putatively functional based on gene clusters. Furthermore, bacteriocins identified from *Enterococcus*, *Staphylococcus* and *Streptococcus* species may have activity against clinically relevant strains, due to the presence of putative immunity genes required for expression in a toxin-antitoxin system. Some strains such as *Paenibacillus larvae* subsp. *pulvifaciens* SAG 10367 contained multiple circular bacteriocin gene clusters from different subfamilies, while some strains such as *Bacillus cereus* BCE-01 contained clusters with multiple circular bacteriocin structural genes.

**Conclusions:**

Sequence analysis provided rapid insight into identification of novel, putative circular bacteriocins, as well as conserved genes likely essential for circularisation. This represents an expanded library of putative antimicrobial proteins which are potentially active against human, plant and animal pathogens.

## Background

Circular bacteriocins are part of class I modified [1] ribosomally produced antimicrobial peptides with a covalent peptide bond between the N and C termini [2, 3]. The circularisation of the molecule improves thermostability, pH tolerance and proteolytic resistance [4], under which conditions most other proteins would be denatured or inactivated. Linearising or nicking circular bacteriocins hampers these intrinsic properties as well as causing a significant reduction in anti-microbial potency [5–7]. They have been shown to work by binding to the cell membrane and creating pores, which act as non-selective ion channels causing cell death [8–11]. Receptor molecules binding circular bacteriocins may also be involved, as demonstrated by garvicin ML targeting the maltose ABC transporter [12].

Bacteriocins have many advantages over traditional antimicrobials such as antibiotics. Bacteriocin resistance has been studied but it appears to occur at varying frequencies [13], indicating each bacteriocin should be tested for resistance before clinical/food use. Because bacteriocins are encoded, it means they can be genetically engineered and targeted towards specific organisms [14, 15]. Due to these characteristics, there is also considerable scope for use in anti-spoilage and food-safety applications.

Circular bacteriocins are class I bacteriocins which can be divided into two families, i and ii based on sequence identity [16, 17]. Table 1 shows the list of experimentally confirmed circular bacteriocins and their characteristics. Class I circular bacteriocins are short sequences (58–70 amino acids in length), four (five in the case of AS-48 and BacA) helical segments that enclose a tightly packed hydrophobic core, a saposin fold, no cysteine pairs, and all (except butyrivibriocin AR10) contain a polybasic region involved in binding to target cell membranes [11, 30, 34, 35].

**Table 1:** Information about the 20 experimentally confirmed circular bacteriocins and their producer strains. ^P^ denotes the characteristic is putative based on the shared characteristics with the other circular bacteriocins

Class	Circular bacteriocin	Bacterial producer	3D structure	α helices	aa size of mature peptide (Mass in Da)	Cluster location	Conjugative plasmid (Y/N/Unknown)	Genes in cluster	Translational coupling	Accession number	Source
i	Aureocyclicin 4185	*Staphylococcus aureus* 4185	Model based on carnocyclin A	4^P^	60 (5607 Da)	pRJ101	Unknown (data unavailable)	11	Y	KF836421	[18]
i	Enterocin NKR-5-3B	*Enterococcus faecium* NKR-5-3B	NMR solution structure	4	64 (6316.4 Da)	Unknown (data unavailable)	N	5	Y	LC068607	[11]
i	Amylocyclicin	*Bacillus amyloliquefaciens* FZB42	N/A	4^P^	64 (6382 Da)	Chromosome	N	6	Y	CP000560.1	[19]
i	Amylocyclicin CMW1	*Bacillus amyloliquefaciens CMW1*	N/A	4^P^	64 (6351.59 Da)	Chromosome	N	6	Y	DF836085	[20]
i	AS-48	*Enterococcus faecalis subsp. liquefaciens* S-48	NMR solution structure	5	70 (7150.17 Da)	pMB2	Y	10	Y	AJ438950.1	[3]
i	bacA	*Enterococcus faecalis* 39–5	N/A	5^P^	70 (7150.17 Da)	pPD1	Y	9	Y	D85752	[21]
i	Carnocyclin A	*Carnobacterium maltaromaticum* UAL307	NMR	4	60 (5862 Da)	Unknown (data unavailable)	Unknown (data unavailable)	10	Y	EU624394	[22]
i	Circularin A	*Clostridium beijerinckii* ATCC 25752	N/A	5^P^	69 (6789.05 Da)	Chromosome	N	8	Y	AJ566621.1	[23]
i	Thermocin 458	*Geobacillus stearothermophilus* DSM 458	N/A	5^P^	70 (6933.25 Da)	Chromosome	N	8	Y	NZ_CP016552.1	[24]
i	Garvicin ML	*Lactococcus garvieae* DCC43	PSIPRED and Jpred3 model	4^P^	60 (6007.2 Da)	Chromosome	N	8^P^	Y	NZ_AMQS01000001	[25]
i	Lactocyclicin Q	*Lactococcus* sp. QU 12	Model using PSIPRED	4^P^	61 (6062.8 Da)	Unknown (data unavailable)	Unknown (data unavailable)	Unknown (data unavailable)	Y	AB462499.1	[26]
i	Leucocyclicin Q	*Leuconostoc mesenteroides* TK41401	N/A	4^P^	61 (6133.23 Da)	Unknown (data unavailable)	Unknown (data unavailable)	5	Y	AB795997.1	[27]
i	Pumilarin	*Bacillus pumilus* B4107	N/A	5^P^	70 (7083.08 Da)	Chromosome	N	5	Y	NZ_AMDH01000012.1	[28]
i	Uberolysin	*Streptococcus uberis* 42	N/A	5^P^	70 (7043.94 Da)	Chromosome	N	6^P^	Y	DQ650653	[29]
ii	Acidocin B	*Lactobacillus acidophilus* M46	NMR solution structure	4	58 (5621.5 Da)	pCV461	Unknown (data unavailable)	7	Y	KP728900.1	[30]
ii	Butyrivibriocin AR10	*Butyrivibrio fibrisolvens* AR10	N/A	4^P^	58 (5981.5 Da)	Chromosome	N	5	Y	AF076529	[31]
ii	Paracyclicin	*Lactobacillus. paracesei* subsp*. paracasei* JCM 8130/ DSM 5622	N/A	4^P^	58 (5905.75 Da)	pLBPC-1	Y	7^P^	Y	NZ_AP012542.1	[32]
ii	Gassericin A/ Reutericin 6	*Lactobacillus gasseri* LA39/ *Lactobacillus reuteri* LA6	N/A	4^P^	58 (5653.6 Da)	pLgLA39	Y	7	Y	AB436615.1	[2]
ii	Plantacyclin B21AG	*Lactobacillus plantarum* B21	N/A	4^P^	58 (5668 Da)	pB21AG01	Y^P^	7	Y	NZ_CP025732.1	[33]
ii	Plantaricyclin A	*Lactobacillus plantarum* NI326	N/A	4^P^	58 (5572 Da)	pB21AG01-like plasmid	Y^P^	7	Y	NZ_NDXC01000075.1	[4]

Circular bacteriocins are usually produced by a gene cluster or operon consisting of 4–10 genes. The mechanism of circularisation and roles of each gene within clusters have not yet been completely elucidated [17, 36], though annotation and mutagenesis studies have provided insight into this [7, 37]. A pre-peptide encoded by the bacteriocin structural gene is produced, followed by signal sequence/leader peptide cleavage. This mature peptide is then able to be either circularised within the cell then secreted which has been shown for leucocyclin Q [38], or secreted and then circularised [39]. The genes involved and the process are not well understood, and it’s possible that different pathways exist for different circular bacteriocins. Circularisation appears contingent on hydrophobic N and C termini residues along with the signal sequence, which is required for correct mature peptide processing [40].

Circular bacteriocin gene clusters are often constituted of overlapping genes, demonstrating a tight organisational structure or genes which depend upon the ribosomal binding site of upstream genes. This indicates expression is regulated by translational coupling [41]. All of the currently identified circular bacteriocin gene clusters contain at least two genes that are translationally-coupled (Table 1).

**Fig. 1:**
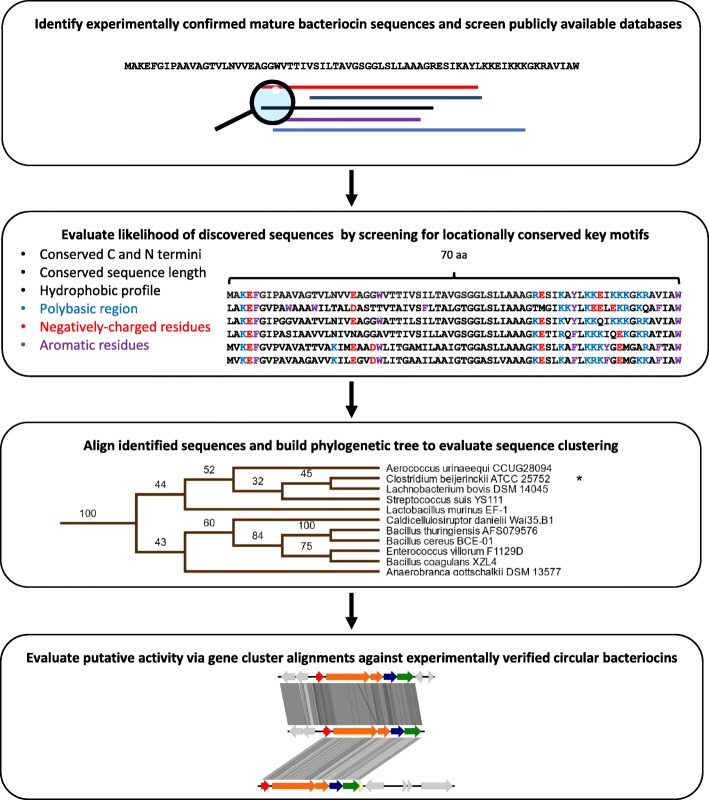
Graphical representation of the analysis workflow leading from sequence acquisition to the classification and evaluation of discovered putative circular bacteriocins

There are twenty experimentally confirmed circular bacteriocins. Evolutionary-based approaches such as sequence alignments, phylogenetics and gene cluster analysis can provide insight and allow novel identification. This study has identified many new and unmentioned putative circular bacteriocins based on sequence similarity from publicly available sequence data. These putative circular bacteriocins were analysed for characteristics commonly found in circular bacteriocins. Figure 1 shows the workflow detailed in this study.

## Results

### Identification and characteristics of putative circular bacteriocins

**Fig. 2:**
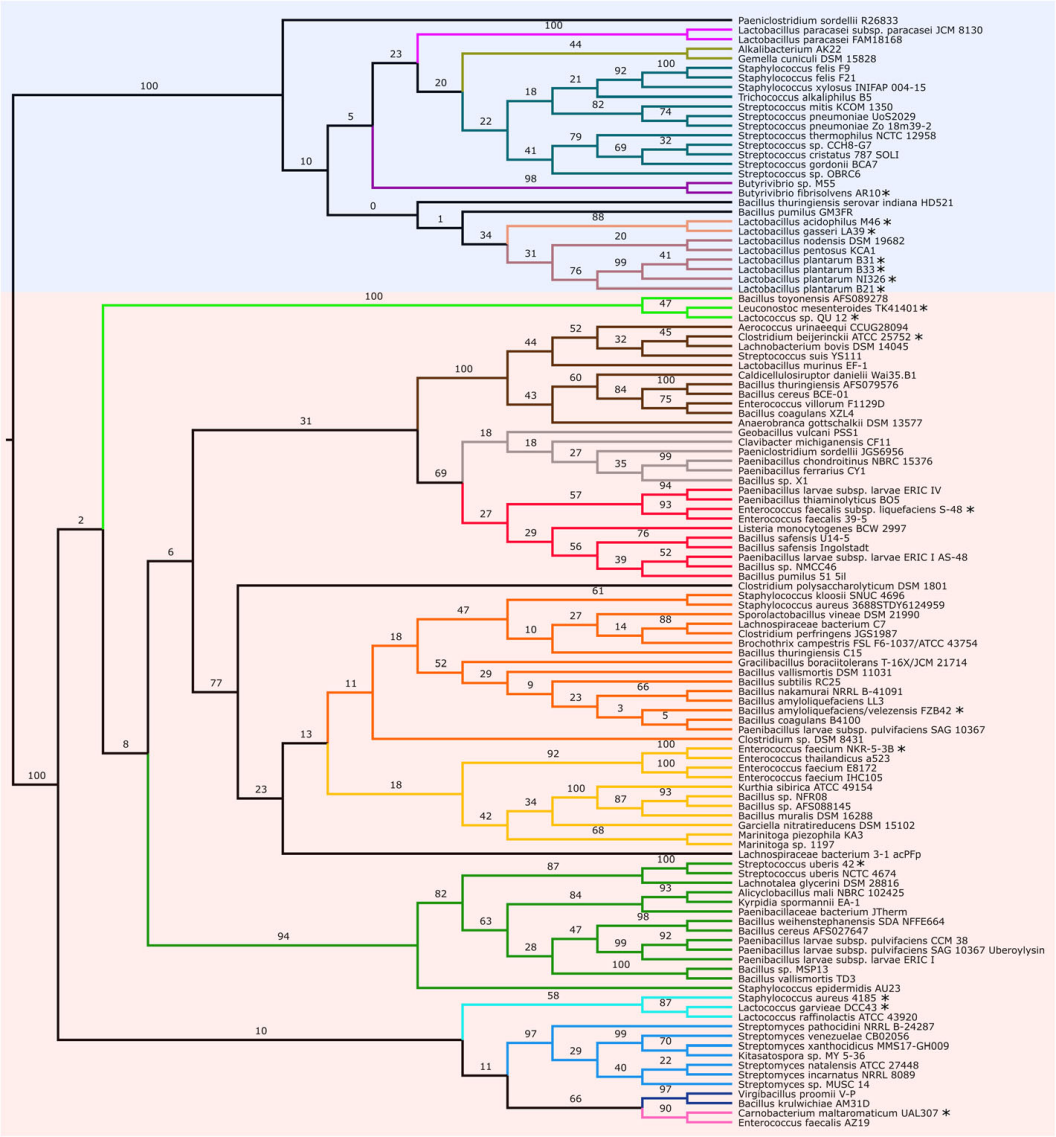
Phylogenetic tree showing the subfamilies of circular experimentally confirmed and putative bacteriocins. *Experimentally-confirmed. Transparent red overlay shows family i. Transparent blue overlay shows family ii. Red: AS-48 subfamily. Orange: amylocyclicin subfamily. Yellow: enterocin NKR-5-3B subfamily. Green: uberolysin subfamily. Light: aureocyclicin 4185/garvicin ML subfamily. Blue: putative venezuelacyclin subfamily. Dark blue: putative krulwicyclin subfamily. Pink: carnocyclin subfamily. Fuschia: paracyclicin subfamily. Gold: alkalicyclin subfamily. Teal: streptocyclin subfamily. Purple: butyrivibriocin AR10 subfamily. Peach: gassericin A/acidocin B subfamily. Brown: plantaricyclin/plantacyclin subfamily. Light green: lactocyclin/leucocyclin subfamily. Dark brown: circularin family. Grey: putative bacillocyclin subfamily. Black shows any putative circular bacteriocins which are too distinct to fit within the proposed subfamilies. *Geobacillus stearothermophilus* DSM 458 not used in phylogenetic tree

This study has identified ninety-nine putative circular bacteriocins within a range of microorganisms. Bringing the total known circular bacteriocins to 119 (Fig. 2, Fig. S1). Five of these have been previously bioinformatically identified [30] but were included in the analyses regardless. Figure S1 contains detailed information about each identified circular bacteriocin, characteristics, strain information and accession numbers. As signal sequences can be highly species specific [11, 42], they were not used for identification of putative circular bacteriocins. Signal sequences are essential for correct folding, circularisation and bioactivity of circular bacteriocins [40]. By removing them from database mining identification of distantly-related putative circular bacteriocins was based on functional antimicrobial protein sequence, rather than irrelevant signal sequence. While some putative circular bacteriocins were annotated correctly, many were unannotated or annotated as branched-chain amino acid aminotransferases which are involved in amino acid catabolism [43], despite having high similarity and sequence motifs to the mature sequences of known circular bacteriocins.

None contained disulphide bonds. Cysteine residues existed only as single residues in 10/119 of the putative and experimentally confirmed sequences, indicating they are not present for disulphide bond formation (Fig. S1). Almost every putative and experimentally-confirmed circular bacteriocin contained a polybasic region. The paracyclicin and butyrivibriocin AR10 subfamilies were distinct from this trend, as they contained 1–2 basic residues. Both paracyclicin and butyrivibriocin AR10 have been experimentally confirmed. The circular bacteriocin from *Alkalibacterium* AK22 (NZ_JANL01000003.1) did not contain any basic residues. 96.6% of the sequences identified contained aromatic residues, which were locationally-conserved (Fig. S1, Table 3). Only *Bacillus krulwichiae* AM31D, *Virgibacillus proomii* V-P and *Alkalibacterium* AK22 contained circular bacteriocin sequences without aromatic residues.

Two *Paenibacillus larvae* strains each harboured two independent putative circular bacteriocins clusters. *Paenibacillus larvae* subsp. *pulvifaciens* SAG 10367 (NZ_CP020557) contained amylocyclicin-like and uberolysin-like clusters, while *Paenibacillus larvae* subsp. *larvae* ERIC_I (NZ_CP019651.1) harboured AS-48-like and uberolysin-like clusters.

Table 2 shows the list of bacteriocins identified which may be active against the WHO’s global priority list of antibiotic resistant bacteria due to the presence of putative immunity genes within the gene clusters [44].

**Table 2:** List of circular bacteriocins identified with potential activity against clinically relevant isolates as part of the WHO’s Global priority list of antibiotic-resistant bacteria to guide research, discovery, and development of new antibiotics [44]

Identified previously/in this study	Pathogen potentially susceptible	Bacteriocin producer	Experimental anti-bacterial activity against clinically relevant bacteria	Source
Identified previously	*Enterococcus faecium*	*Enterococcus faecalis* 39–5	*S. aureus, E. faecalis, E. faecium, S. agalactiae, S. sanguis*	[45]
	*Enterococcus faecium*	*Enterococcus faecalis* subsp. *liquefaciens* S-48	*S. faecalis* S-47, *Escherichia coli* U-9, *E. faecalis* OGlX	[46, 47]
	*Enterococcus faecium*	*Enterococcus faecium* NKR-5-3B	*B. coagulans, B. subtilis, L. lactis* ssp. *lactis*, *L. sakei* ssp. sakei	[48]
	*Staphylococcus aureus*	*Staphylococcus aureus* 4185	*B. cereus, B. coagulans, B. licheniformis, L. monocytogenes, M. luteus*	[49, 50]
	*Streptococcus pneumoniae/*Group A/Group B	*Streptococcus uberis* 42	*E. faecalis, E. hirae, M. luteus, S. aureus, S. agalactiae, S. salivarius, S. pyogenes, S. equisimilis, S. dysgalactiae, S. anginosus*	[29]
Identified in this study	*Enterococcus faecium*	*Enterococcus villorum* F1129D	N/A	This study
	*Enterococcus faecium*	*Enterococcus thailandicus* a523	N/A	This study
	*Enterococcus faecium*	*Enterococcus faecium* E8172	N/A	This study
	*Enterococcus faecium*	*Enterococcus faecium* IHC105	N/A	This study
	*Enterococcus faecium*	*Enterococcus faecalis* AZ19	N/A	This study
	*Staphylococcus aureus*	*Staphylococcus felis* F9	N/A	This study
	*Staphylococcus aureus*	*Staphylococcus felis* F21	N/A	This study
	*Staphylococcus aureus*	*Staphylococcus xylosus* INIFAP 004–15	N/A	This study
	*Staphylococcus aureus*	*Staphylococcus kloosii* SNUC 4696	N/A	This study
	*Staphylococcus aureus*	*Staphylococcus aureus* 3688STDY6124959	N/A	This study
	*Staphylococcus aureus*	*Staphylococcus epidermidis* AU23	N/A	This study
	*Streptococcus pneumoniae/*Group A/Group B	*Streptococcus uberis* NCTC 4674	N/A	This study
	*Streptococcus pneumoniae/*Group A/Group B	*Streptococcus suis* YS111	N/A	This study
	*Streptococcus pneumoniae/*Group A/Group B	*Streptococcus* sp. OBRC6	N/A	This study
	*Streptococcus pneumoniae/*Group A/Group B	*Streptococcus gordonii* BCA7	N/A	This study
	*Streptococcus pneumoniae/*Group A/Group B	*Streptococcus cristatus* 787_SOLI	N/A	This study
	*Streptococcus pneumoniae/*Group A/Group B	*Streptococcus* sp. CCH8-G7	N/A	This study
	*Streptococcus pneumoniae/*Group A/Group B	*Streptococcus mitis* KCOM 1350	N/A	This study
	*Streptococcus pneumoniae/*Group A/Group B	*Streptococcus pneumoniae* UoS2029	N/A	This study
	*Streptococcus pneumoniae/*Group A/Group B	*Streptococcus thermophilus* NCTC 12958	N/A	This study
	*Streptococcus pneumoniae/*Group A/Group B	*Streptococcus pneumoniae* Zo 18 m39–2	N/A	This study

### Phylogenetics of circular bacteriocins

Based on the sequence analysis of bacteriocins, there appears to be two different families of class I circular bacteriocins, family i and ii, each cluster with bootstrap values of 100 [16, 17] (Fig. 2, Fig. S2). Out of the 119 sequences, 89 (74.8%) are part of family i while 29 (24.4%) are from family ii (Fig. S2, Table 3). However, there is considerable sequence divergence within these families, with family i demonstrating a wide variety of sequence lengths and compositions. Therefore, the most appropriate way to classify these sequences was to separate them based on their most closely-related experimentally confirmed circular bacteriocin. In some cases such as streptocyclin, divergence was considered too high (based on bootstrap values) and new subfamilies were coined using the ‘cyclin’ suffix.

**Table 3:** Summary results of the mature bacteriocin sequence and gene cluster analysis

	Characteristic	No. of circular bacteriocins	Percentage of bacteriocins (%)
**Mature bacteriocin sequence**	Total circular bacteriocins	119	100.0
	Experimentally-confirmed	20	16.8
	Total identified in this study	99	83.2
	Containing polybasic region	111	93.3
	Containing aromatic residues	115	96.6
	Family i	90	74.8
	Family ii	29	24.4
**Bacteriocin cluster**	Predicted functional (conservative)	90	75.6
	Translational coupling	109	91.6
	Chrosomosomally-located	77	64.7
	Plasmid-associated	25	21.0
	Unknown location	16	13.4
	Mobile genetic element-associated	24	20.2

Due to phylogenetic ambiguity and divergence of the identified circular bacteriocin sequences, it was inappropriate to classify each putative circular bacteriocin into currently identified/characterised subfamilies. To remedy this, new circular bacteriocin subfamilies were proposed and named including streptocyclin, akalicyclin, krulwicyclin, bacillocyclin and venezuelacyclin (Fig. S1).

Family i was composed of the circularin, lactocyclin/leucocyclin, bacillocyclin, AS-48, amylocyclin, enterocin NKR-5-3B, uberolysin, aureocyclicin 4185/garvicin ML, venezuelacyclin, krulwicyclin and carnocyclin A subfamilies. Family ii were composed of the paracyclicin, akalicyclin, streptocyclin, butyrivibriocin AR10, gassericin A/acidocin B and plantaricyclin/plantacyclin subfamilies. Due to sequence similarity and phylogenetic branch position, several experimentally confirmed circular bacteriocins were classified within the same subfamily. They included aureocyclicin 4185 and garvicin ML (61.4% similarity), lactocyclin and leucocyclin (82% similarity), gassericin A and acidocin B (100% similarity), plantaricyclin and plantacyclin (94.8% similarity). Some of these subfamilies will most likely fracture into clearer, distinct subfamilies as more sequences become available. Several putative circular bacteriocins were found on lone phylogenetic branches and did not fit into subfamilies and were not classified beyond the familial level.

### Hydrophobicity of mature circular bacteriocins

**Fig. 3:**
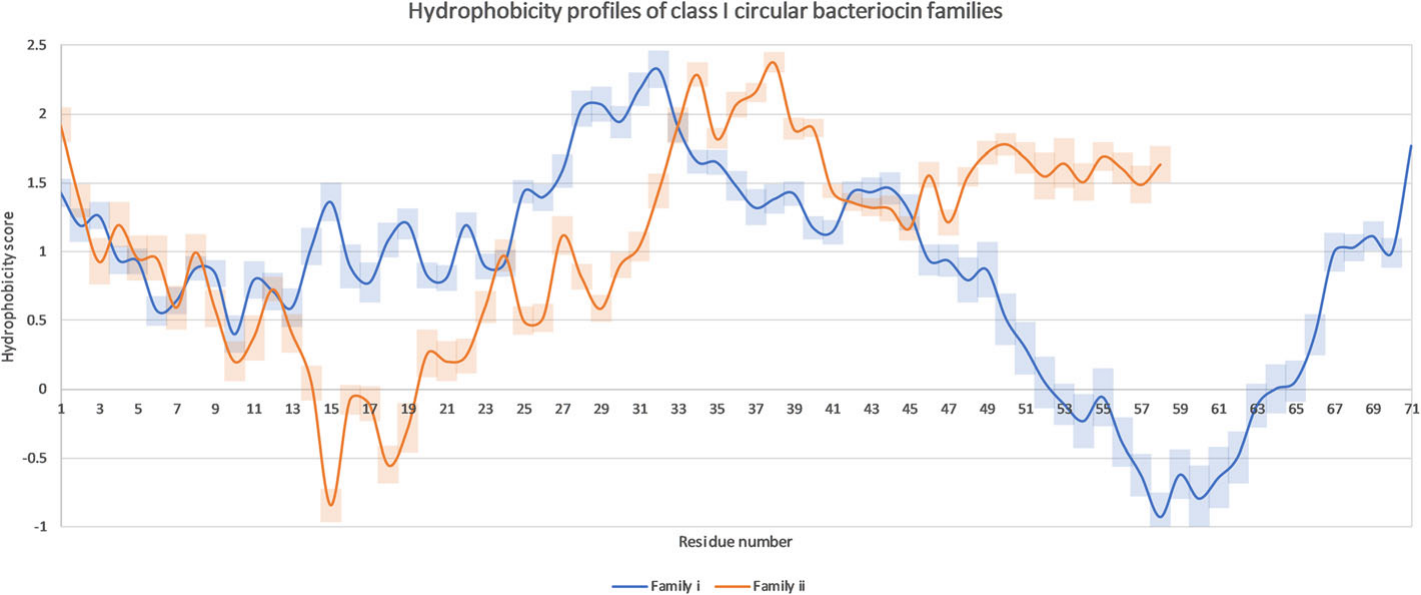
Hydrophobicity profiles of two circular bacteriocin families. The blue lines shows family i and the orange line shows ii. The semi-transparent bars show the 95% confidence intervals. Hydrophobicity scores were calculated based on Kyte and Doolittle with a sliding window of 9 [51]

Analysis of hydrophobicity profiles suggested two major profiles (Fig. 3), with a few exceptions (Fig. S3). This gave further evidence that the putative sequences identified were most likely circular bacteriocins. The two major hydrophobicity profiles of the circular bacteriocins matched the phylogenetic family classifications of family i and ii (Fig. 3). It appears, despite sequence divergence within families, residues are mutating to residues which maintain the hydrophobic profile of the protein. In general, the N terminus of class I i tended to have a variable hydrophobic profile, reflecting the sequence divergence and residue length differences within the family.

Both families have similar regions within the hydrophobicity profiles, despite the sequence variability within and between them. In general they are considerably hydrophobic. The C and N termini of every sequence was also found to be hydrophobic (Fig. S3). Both families also have a notable polybasic region (residues 52–65 in family i and 14–19 in ii) which produces two hydrophilic troughs.

Despite not fitting into any direct phylogenetic subfamilies within family ii, *Bacillus pumilus* GM3FR, *Paeniclostridium sordellii* R26833 and *Bacillus thuringiensis* serovar *indiana* HD521 all match the hydrophobic profile of family ii. Sequence logos (Fig. S4) showed high levels of conservation within the ii family, while i had high levels of conservation at the N and C termini. The conserved termini may be implicated as a ligation motif, allowing circularisation of the C and N termini.

### Gene cluster analysis

To determine the number of putatively functional circular bacteriocins, each putative cluster was compared to the cluster of its most closely related experimentally confirmed circular bacteriocin (Fig. S5). A cluster was determined ‘putatively functional’ if it contained matching genes required for circular bacteriocin production of its phylogenetically closest relative. Table 3 shows a summary of this analysis.

Though there was high cluster divergence between families, similar genes were found in clusters in almost every case, but not limited to: ABC transporters, putative immunity gene/s, transmembrane proteins, SpoIIM proteins, permeases etc. Table S1 shows a general summary across the identified subfamilies. This provided more evidence that most of these putative sequences were circular bacteriocins, in line with the sequence similarity and hydrophobic profile results. Different gene clusters showed different degrees of similarity, with many having gene rearrangements, inversions, insertions and sharing low sequence similarities between homologues.

Several bacteriocin clusters appeared incomplete (Fig. S5) and it is probable that some of these clusters were vestigial or pseudogenes. Of the total 119 circular bacteriocin clusters, a conservative estimate of 90 (75.6%) were putatively functional (Fig. S1), though the number is likely higher due to the percentage of gene clusters which contain translational coupling (91.6%). As this analysis was restricted by limited sequence data and assemblies, other genes outside the clusters required for circular bacteriocin production may be present elsewhere in the genome. These would be functional but would be scored as non-functional via this analysis. ABC transporters were seen in every single experimentally confirmed circular bacteriocin cluster, as well 95/99 of putatively identified clusters (Figs. S1, S5). This indicates that these 4 circular bacteriocins without ABC transporters were either inactive vestigial remnants or exported via another ABC transporter. Circular bacteriocin ABC transporters are highly similar to ABC transporters within the genomes. It was unclear if non-cluster transporters would be involved in production of circular bacteriocins and were thus considered putatively non-functional. HlyD-like and efflux RND transporters were only present in a few clusters within subfamilies and were not indicative of a putatively functional cluster, as previously demonstrated [52]. The clusters from *C. polysaccharolyticum* DSM 1801 and *L. bacterium* 3–1 acPFp are examples of unambiguously disrupted gene clusters which would most likely be non-functional. The summary of the cluster analysis for each putative circular bacteriocin (functional/non-functional) is found in (Fig. S1 and Table 3).

21% of the clusters were found on plasmids, 64.7% were chromosomally located, and the remaining 13.6% were considered unknown (Table 3). 20.2% were associated with mobile genetic elements such as insertion sequences (Fig. S5).

**Fig. 4:**
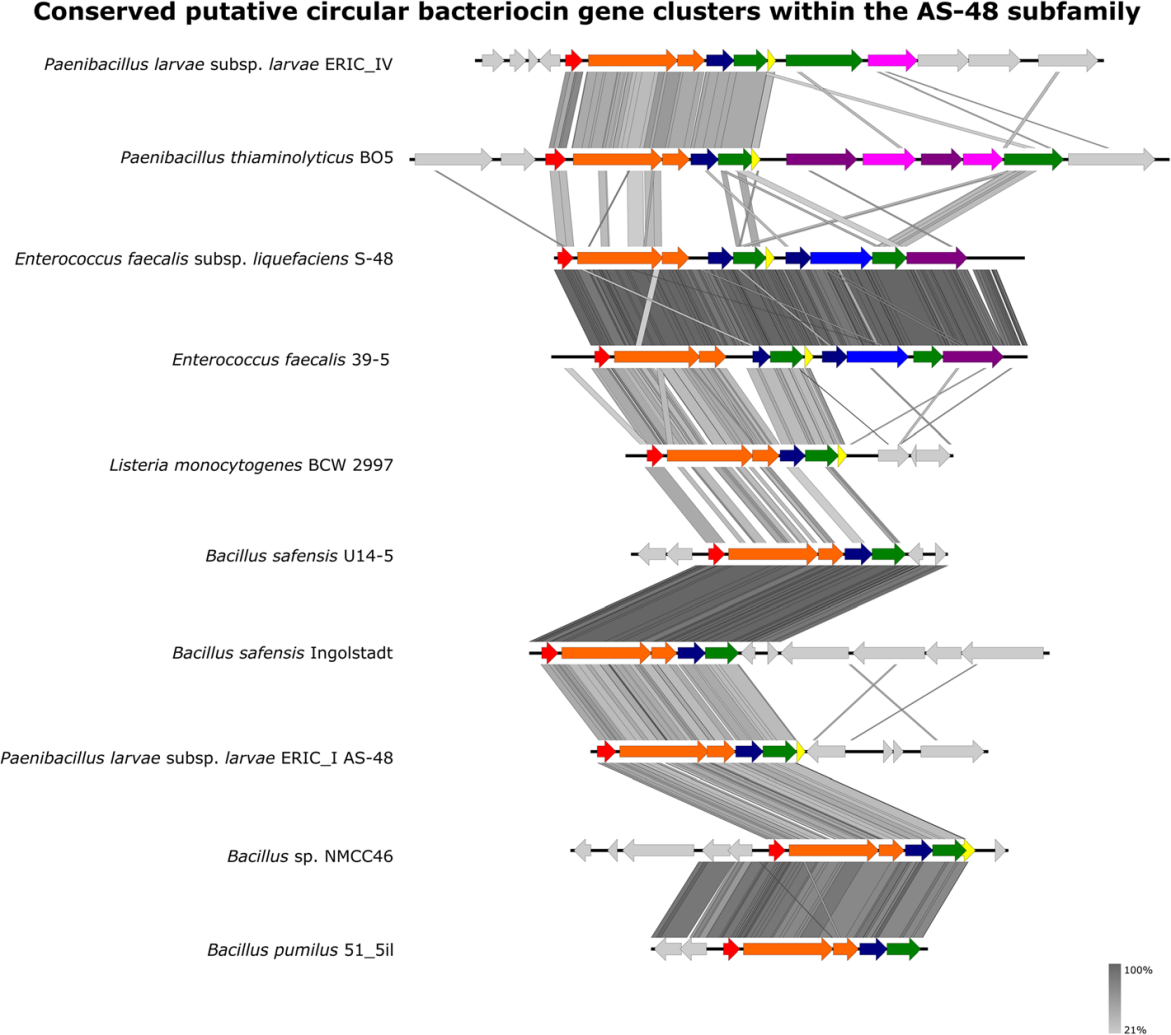
Diagram showing the conserved genes within the AS-48 subfamily. Arrows show putative genes within the gene cluster. Red shows the bacteriocin structural gene. Orange shows putative membrane proteins/stage II sporulation protein M. Yellow shows putative immunity genes such *as-48D1*. Green shows putative ABC-transporter proteins such as *as-48D* and *as-48G*. Blue shows HlyD/efflux RND transporters such as as-48F. Dark blue shows other transmembrane proteins. Pink shows putative binding-protein-dependent proteins and extracellular solute-binding proteins. Purple shows ABC-II/FtsX permeases such *as-48H*, based on work done with AS-48 [53]. Grey shows genes of unknown function which may or may not be related to circular bacteriocin production. Figure produced in Easyfig and Inkscape

In the AS-48 subfamily (Fig. 4), six genes *as*-*48ABCC1DD1* have been shown to be essential for AS-48 production [52]. This consists of the bacteriocin structural gene, a short and long putative membrane protein/stage II sporulation protein M, another putative transmembrane protein, an ABC transporter and an immunity gene [52]. All six genes were found in most clusters, though putative immunity genes were not identified in 3/10 clusters. This analysis revealed stage II sporulation protein M domains were commonly found in the putative membrane proteins of the identified circular bacteriocin clusters. Other times, they were found encoded by two separate genes (Fig. 4). Therefore, they were treated as similar genes.

Immunity genes from clusters of experimentally confirmed circular bacteriocins appear to have two to three transmembrane domains (Fig. S3). They also contain large hydrophilic region/s which occur between these domains. Acidic residues were also found outside these transmembrane domains in 10/15 experimentally-confirmed circular bacteriocin immunity genes. There were no cysteine pairs found in the immunity genes except for in the atypical lycD sequence from leucocyclicin Q.

To demonstrate the identification of putative circular bacteriocin subfamilies, which were most likely functional, cluster analysis of the putative bacillocyclin subfamily is shown in Fig. S6. Five of the six gene clusters match the gene cluster profile of the AS-48 subfamily (closest phylogenetic relative) and appear to be intact.

**Fig. 5:**
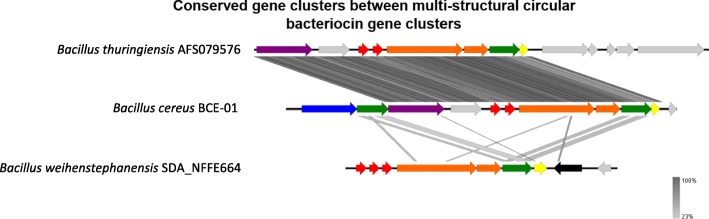
Diagram of circular bacteriocin gene clusters with multiple structural genes. Arrows show putative genes within the gene cluster. *B. weihenstephanensis* SDA_NFFE664 contains three structural genes spaced with independent promoters, while *B. cereus* BCE-01 and *B. thuringiensis* AFS079576 contain two. Red shows bacteriocin structural genes. Orange shows putative membrane proteins/stage II sporulation protein M. Yellow shows putative immunity. Green shows putative ABC-transporter proteins. Blue shows HylD/efflux RND transporters. Purple shows ABC-II/FtsX permeases. Black shows mobile genetic elements such as phage proteins/transposons/IS elements. Grey shows genes of unknown function which may or may not be related to circular bacteriocin production. Figure produced in Easyfig and Inkscape

Another previously undescribed observation was that some strains contained multiple structural bacteriocin genes within the same cluster (Fig. 5). *Bacillus cereus* BCE-01 (NZ_MVPV01000042.1) contained two different circularin-like circular structural bacteriocin genes with 82.89% identity. 80% identity was found between the signal sequences of these two structural genes. *Bacillus thuringiensis* AFS079576 (NZ_NUXU01000032.1) also contained two circularin-like structural genes with 81.58% identity within the same cluster. 80% identity was found between the signal sequences of these two structural genes. *Bacillus weihenstephanensis* SDA_NFFE664 (NZ_FMBF01000026.1) contained three uberolysin-like circular structural genes with 100% identity and 92% identity, respectively. Each circular bacteriocin structural gene from *B. weihenstephanensis* SDA_NFFE664 had identical signal sequences to the others in the cluster.

Each structural gene within these multi-structural gene clusters had independent putative promoters. Another observation is that a single putative immunity gene was found within these clusters, indicating it is most likely the single immunity factor for each circular bacteriocin variant.

## Discussion

### Putatively functional circular bacteriocins

This study shows that circular bacteriocins are much more prevalent than originally expected [39]. Previous bioinformatics efforts have identified uncharacterised novel circular bacteriocins [28, 30]. Through the use of the mature sequences only, we have identified a large number of previously undescribed putatively circular bacteriocins. This approach differs from BAGEL which also includes signal sequences [54]. Although no sequence is publicly available, a circular bacteriocin was likely isolated from *Lactobacillus acidophilus* IBB 801 [55]. Some circular bacteriocins identified here have 100% similarity to other circular bacteriocins despite being present in different species. This study has shown bacteria from a wide range of sources including milk, soil, urine, plant cores, honeybee larvae, deep sea water and more (Fig. S1), contain putatively functional circular bacteriocin clusters. This indicates a potentially large reservoir of circular bacteriocin-producing strains and circular bacteriocins which could be used as therapeutics, food preservatives [39], or in other applications such as use as vector proteins to stabilise bioactive proteins [56]. There are many bioactive peptides which report low stability [57], which could be stabilised with C-N terminal ligation [58] found in circular bacteriocins. During the process of this manuscript being written, circular bactercion amylocyclicin CMW1 was discovered [20]. This sequence was successfully predicted as a circular bacteriocin from this dataset, appearing in *Bacillus amyloliquefaciens* LL3. This co-occurrence provides more evidence that the predicted circular bacteriocins are likely correctly identified.

*Bacillus* spp. also contained the largest range of putative circular bacteriocins in this dataset. They contained clusters from family i subfamilies: AS-48, amylocyclicin, enterocin NKR-5-3B, uberolysin, lactocyclin/leucocyclin, circularin, bacillocyclin, krulwicyclin. They also contained family ii circular bacteriocins which were not assigned subfamilies. However, this may have been due to their phylogenetic heterogeneity, some of which has been remediated though reclassifications based on next generation sequencing rather than phenotype [59, 60].

The percentage of gene clusters which contain translational coupling (91.6%) is most likely a better representation of functional clusters than the conservative prediction based on gene presence (75.6%) found in Table 3. Translational coupling indicates a high level of cluster structure conservation [61] and it would be highly unusual for these genes to be asserting such a high degree of organisational structure if they were not positively-selected for, that is if they were not functional/expressed. Mutations in these tightly-packed clusters will not only alter the ends of particular gene products, but also impact transcription of downstream genes in alternative reading frames.

Presence of polybasic and aromatic residues were locationally conserved, found in 93.3 and 96.6% of identified circular bacteriocins, respectively (Table 3, Fig. S1). Aromatic residues are often found flanking transmembrane-associated helices, allowing penetration into membranes [62, 63]. Trp24 has been shown to be essential in the biological activity of AS-48, as it is located in a hydrophobic region that interacts with the membrane [64].

It has been previously pointed out that circular bacteriocins have similar hydrophobicity profiles [5]. Analysis of hydrophobicity profiles allowed increased confidence in the identification of putative circular bacteriocins discerned through sequence similarity. Hydrophobic profiles were maintained within subfamilies, as well as more generally within the families i and ii. By comparing profiles of putatively identified sequences to the average profile of each family, it can be determined which family they belong to. This could also be used to screen out non-circular bacteriocins. Though the hydrophobicity profiles are different between the families i and ii, if the profile of ii is flipped, the profile is surprisingly similar to family i (Fig. S3). There are particular sequences which show divergence to the profiles, such as *L. mesenteroides* TK41401 (leucocyclicin Q) and *Lactococcus* sp. QU 12 (lactocyclicin Q) from family i, and *Trichococcus alkaliphilus* B5 (paracyclicin subfamily) and *Alkalibacterium* AK22 (akalicyclin subfamily) from ii.

A hydrophilic region was found in every putative and experimentally confirmed circular bacteriocin (Fig. 3, Fig. S3). This usually overlapped with the uni/polybasic region and implied a conserved functional region. There is strong evidence for a similar mechanism of action for this region, given the high levels of evolutionary conservation. This region is most likely involved with cell membrane interaction and binding based on the positively-charged basic residues and the negatively-charged cell membrane [65, 66]. In experiments, the positively-charged (and polybasic) region of AS-48 (residues 49–69) showed no killing activity, but showed competitive binding to the negatively-charged membrane against the wild type AS-48 bacteriocin [65], indicating the role this region plays in the bactericidal activity of circular bacteriocins. Butyrivibriocin AR10 uncharacteristically does not contain a polybasic region (only a single basic residue), yet is functional as a circular bacteriocin against other *B. fibrisolvens* isolates [67]. It has a hydrophobic profile with a hydrophilic region which is consistent with family ii. This indicates polybasic regions aren’t necessarily required for antimicrobial activity, but the hydrophilic region is.

### Phylogenetics

Phylogenetic classification has resolution trade-offs. A higher number of families (reasonably up to 6) could have been attributed, though due to the similar proposed modes of action [17, 39] and conserved structural motifs [11, 30, 34, 35], further familial division would result in diminished returns. By classifying circular bacteriocins into groups with higher resolution such as subfamilies, experimentally-confirmed circular bacteriocins can be used as type-sequences and accurate sequence analysis and comparisons can be performed. This reduces the background noise of distantly-related circular bacteriocins within the immediate sequence family. It is highly probable that the putative circular bacteriocins within each subfamily share a similar mechanism of action but have their own distinct spectrum of activity. The phylogenetic classifications were further enforced by cluster analysis. For example, uberolysin and amylocyclicin circular bacteriocin subfamilies are distinct at the cluster level, have different hydrophobicity profiles at their C termini (Fig. S3), yet are not divergent regarding structural gene homology despite a size difference of 6 residues.

### Conserved genes within circular bacteriocin clusters

Cluster analysis proved to be informative for determining putative functional circular bacteriocins, as well as phylogenetic classification. Recently-diverged structural genes would most likely have similar associated genes within their bacteriocin clusters. The drawback of this type of analysis was the associated genes essential for circular bacteriocin product may not be present within the same cluster but elsewhere within the genomic material. However, given a conservative 75.6% estimate of putative functionality, a number of potentially useful antimicrobial peptides have been highlighted. It is probable that some of these clusters contain non-functional pseudogenes, but given that most clusters were ‘intact’ upon comparison to experimentally confirmed clusters, the genes are considered conserved for circular bacteriocin production [2, 4, 18, 19, 21–23, 25–27, 29–33, 38, 41, 52, 68, 69].

Stage II sporulation protein M membrane proteins were found in almost every identified cluster, indicating they are an essential gene and their absence was considered for putative cluster functionality. This observation has been previously observed [70]. SpoIIM proteins likely form a protein-protein complex with the ABC transporter, acting as the membrane spanning domain as the ABC transporter proteins do not contain any transmembrane domains. Mature circularised bacteriocin accumulated within cells when the DUF95 superfamily protein (SpoIIM) was removed within leucocyclicin Q gene cluster [38]. This indicates post-translational circularisation occurs internally before export. The ABC transporter was unable to secrete the bacteriocin as it was not anchored at the membrane.

Strains with multi-structural gene clusters are an undescribed phenomenon until now. Given their high sequence identities to each other, it is clear they are a result of duplication events in which slight variants with independent promoters have been selected for. It is most likely that these strains swap or co-express variable circular bacteriocins via response regulators and quorum sensing [29, 31, 71], allowing expression of different circular bacteriocins with a slightly different spectrum of activity/microbial targets. These multi-structural gene clusters can also give us insights into the putative immunity genes. It appears one putative immunity gene is enough to provide protection against each circular bacteriocin variant within the cluster. This indicates immunity genes may provide broader immunity than once thought and may possibly provide immunity to similar circular bacteriocins with as low as ~ 80% similarity. Based on the presence of two (sometimes three) putative transmembrane domains, as well as the central hydrophilic region and presence of acidic residues at the termini, the mechanism of immunity can be proposed. Immunity proteins may function as transmembrane proteins and competitively bind positively-charged/polybasic regions of corresponding circular bacteriocins, thus reducing pore formation within the cell membrane. Acidic residues found in the immunity proteins may compete with the negatively-charged cell membrane. Though, further experimental analysis is required, as immunity has been shown to be a cumulative effect with other genes within the cluster demonstrating a role in immunity [38, 41, 53]. More broadly, the observation that immunity genes are present in most gene clusters indicates these bacteria are susceptible to their own bacteriocins. Therefore, related species may also prove susceptible if lacking the corresponding immunity gene. This is hopeful as circular bacteriocins identified here were found in *Enterococcus*, *Staphylococcus* and *Streptococcus* species, which are currently regarded by the WHO as priority organisms for discovery of new antimicrobials [44].

### Selfish genetic elements

Although providing fitness to the cell, circular bacteriocins and their associated clusters can be thought of as selfish genetic elements. Given the high stability of circular bacteriocins, if at any time the cluster is mutated or plasmid is lost, the immunity factors associated with the cluster may also be lost. The ex-producer would then be susceptible to the bacteriocin, and therefore this phenotype will be selected against. Also, given the high temporal stability of circular bacteriocins, they would also be more stable than the immunity genes which would be more susceptible to proteases, heat, pH etc., and would require continual renewal via gene expression. By nature, it is a toxin-antitoxin system which locks the producing strain into a long-term partnership. It has been demonstrated by removing the circular bacteriocin gassericin A from a plasmid, segregational stability of that plasmid drops [72]. This explains why so many of the circular bacteriocin clusters identified were putatively intact (Table 3), regardless if they are chromosomally associated or plasmid-borne. As previously described, the spectrum of antimicrobial activity (usually to closely related species) of circular bacteriocins provides further evidence of the toxin-antitoxin relationship [22, 24, 72]. Coincidentally, the circular bacteriocin from *L. nodensis* DSM 19682 was previously highlighted by a similar genome-mining study and the strain was not found to demonstrate antimicrobial activity against a range of bacteria including *Enterococci* and *Lactobacilli* [32]. Given that the gene cluster was identified as intact (Fig. S1, Fig. S5), it is possible the bacteriocin was not tested against closely-related strains (including *L. nodensis*) which may demonstrate susceptibility.

A circular bacteriocin cluster missing only an immunity gene has several explanations and may still potentially be active despite missing a putative immunity gene (though not considered ‘putatively active’ in this study). Production of the circular bacteriocin without immunity factors generally results in self-killing [4]. The first explanation is that the bacteriocin is not functionally expressed. Alternatively, if the antimicrobial activity mode of action relies on specific target receptors not found in the producer strain as in the case of Garvicin ML [12], immunity genes would not be needed, as lacking the target gene would be enough to confer immunity. Another alternative explanation is recent inactivation of the entire immunity-gene-lacking cluster, which given enough time will eventually be reduced to pseudogenes and vestigial fragments. Being associated with conjugative plasmids or mobile genetic elements (Table 3) such as transposons allows wider dissemination of these genes within populations.

## Conclusions

This work has identified 94 novel and previously undescribed circular bacteriocins utilising known translated DNA sequences of mature bacteriocins. A small number of these sequences have been previously described by bioinformatic approaches [28], however other sequences identified in this work were either incorrectly annotated in publicly available databases or not annotated at all. All sequences were found in Gram positive bacteria. Phylogenetic analysis allowed clustering of these bacteriocins into two families (i and ii) which is consistent with previous literature. To further evaluate these bacteriocin sequences as legitimate, sequences were classified into subfamilies based off sequence similarity to experimentally confirmed circular bacteriocins. These subfamilies were confirmed by comparing the bacteriocin gene clusters of experimentally-confirmed circular bacteriocins. The cluster analysis was highly consistent with the mature bacteriocin sequence phylogeny clustering. Almost all gene clusters were also found to have translational coupling. This analysis was also able to demonstrate the minimum essential genes required for circular bacteriocin production and secretion, allowing identification of putatively active bacteriocin clusters. Classification of the bacteriocins into subfamilies allowed high resolution sequence analysis which can be used to identify important residues, motifs, inform mutagenesis studies and synthetic design of future circular antimicrobial proteins.

Analysis of the mature bacteriocin sequences revealed several important motifs which were consistent across almost every sequence identified within the two families and within each subfamily. These motifs were locationally consistent within but not between the two families. Motifs include conserved C and N termini within subfamilies, sequence length, consistent hydrophobic profiles (Fig. 3) despite sequence dissimilarity, a polybasic region likely involved in membrane binding and aromatic residues flanking transmembrane-associated helices likely involved in membrane penetration. The presence of these motifs across two independent families of this class of antimicrobial indicates their importance in expression and activity of circular bacteriocins.

Finally, the gene cluster analysis revealed almost every circular bacteriocin cluster contained immunity genes. This indicates the antimicrobial activity is also likely active against the producer strain, as part of a toxin-antitoxin system. This realisation allows genome mining to perform a targeted approach to combat pathogens, namely as the bacteriocins are active against the producer strain. We identified putatively active circular bacteriocin clusters from high priority pathogens *Staphylococcus*, *Streptococcus* and *Enterococcus* species which should be active against clinically relevant strains. Future work should involve the isolation of these producer bacteria (Table 2) and screening their culture supernatants against clinical isolates to characterise and identify these bioinformatically identified antimicrobials.

## Methods

### Identification of putative circular bacteriocins

NCBI was mined (date accessed: 20/2/19) against the 17 known mature circular bacteriocin sequences minus signal sequences.

### Phylogenetic analysis of putative circular bacteriocins

Clustal Omega (https://www.ebi.ac.uk/Tools/msa/clustalo/) (date accessed 25/2/19) [73] was used for alignments and exported to fasta format, which was used as input for RAxML (raxmlHPC-PTHREADS-SSE3 version 8.2.10) [74] using the following parameters for ML + rapid bootstrap analysis with 100 replicates:

-T 2 -f a -× 285 -m PROTGAMMABLOSUM62 -p 639 -N 100.

The bipartitions output file was used in FigTree version 1.4.4 (http://tree.bio.ed.ac.uk/software/figtree/) for viewing/manipulation. Microsoft Excel version 1902 was used to compile the table of putative circular bacteriocin and characteristics, which was then manipulated using Inkscape version 0.92 (https://www.inkscape.org).

### Circular bacteriocin characteristics

Polybasic residues were identified in the mature bacteriocin via the ‘Mark’ function in Notepad++ version 7.5.9 searching for the string “R|K|H” using the following search modifiers: ‘Regular expression’ and ‘Match case’.

### Hydrophobicity analysis

Hydrophobicity profiles were generated using the protscale website https://web.expasy.org/protscale/ with a sliding window of 9 [51]. 95% confidence intervals were calculated using the Descriptive Statistics module from the Data Analysis ToolPak in Microsoft Excel. As C and N termini would be joined in the mature circular bacteriocin form, the first four residues were copied to the end of the sequence and the final four residues were copied to the beginning of the sequence to account for the sliding window of 9. This was performed by searching the amino acid fasta file for: ^(([A-Z]{4})([A-Z]*)([A-Z]{4}))$ and replacing with $4$1$2 with search modifiers: ‘Regular expression’ and ‘Match case’ in Notepad++.

### Transmembrane domain analysis

Sequences were submitted to Phobius [75] (date accessed: 1/3/19).

### Sequence logos

Skylign (date accessed: 9/4/19) was used after Clustal Omega alignments using the ‘Observed Counts’, ‘Alignment sequences are full length’, and ‘Information Content – All’ parameters [76].

### Gene cluster analysis

To determine if the circular bacteriocin structural gene and associated gene clusters were present on plasmids or chromosome, tBlastn and BLASTn (https://blast.ncbi.nlm.nih.gov/Blast.cgi) [77] was used to see if there were significant nucleotide hits to plasmids or chromosomes on NCBI. Size was also considered; if a gene cluster was on a contig > 100 kb, it was considered most likely chromosomal. Functional domains were determined using HMMER version 3.2.1 (http://hmmer.org/) [78], along with NCBI annotations to infer gene function. Presence of plasmid-determinants such as repA/B and mobilisation genes were used to determine presence of cluster on plasmid. Presence of chromosomal determinants such as the 16 s and tRNA genes were used to infer chromosomal localisation. If location was unclear, they were determined as ‘Unknown’.

For gene clusters broken up amongst multiple contigs, contigs containing cluster elements were first joined with 5 N’s, and then used for cluster alignments and analysis.

Easyfig version 2.2.3 [79] was used to align and visualise gene clusters using the tblastx function with an e-value cut-off of 0.001. *Lactococcus* sp. QU 12 was excluded from cluster analysis as only the structural gene sequence data is publicly available.

## Supplementary information

**Additional file 1: Figure S1.** Table containing further, detailed information of each circular bacteriocin. 

**Additional file 2: Figure S2.** Phylogenetic tree showing the two subfamilies of circular bacteriocins. Family i is shown in red, while ii is shown in blue.

**Additional file 3: Figure S3.** Raw data of hydrophobicity scores from each bacteriocin.

**Additional file 4: Figure S4.** Sequence logo generated using Skylign after Clustal Omega alignment. The top logo shows family i. The bottom logo shows family ii. Family i has a sequence length of 84 due to the sequence variation within the family, resulting in a gapped alignme.

**Additional file 5: Figure S5.** Series of figures showing the gene cluster analysis.

**Additional file 6: Table S1.** Summary of circular bacteriocin gene clusters and their general distribution throughout the circular bacteriocin subfamilies. Not every circular bacteriocin cluster within each subfamily was predicted to contain each of the genes here, though generally this was the case.

**Additional file 7: Figure S6.** Bacillocyclin subfamily cluster analysis. Diagram showing the conserved genes within the putative bacillocyclin subfamily. Arrows show putative genes within the gene cluster. Red shows the bacteriocin structural gene. Orange shows putative membrane proteins/stage II sporulation protein M. Yellow shows putative immunity genes such *as-48D1*. Green shows putative ABC-transporter proteins such as *as-48D* and *as-48G*. Blue shows HylD/efflux RND transporters such as as-48F. Dark blue shows other transmembrane proteins. Pink shows putative binding-protein-dependent proteins and extracellular solute-binding proteins. Purple shows ABC-II/FtsX permeases such *as-48H*, based off the work done with AS-48 [4]. Grey shows genes of unknown function which may or may not be related to circular bacteriocin production. Figure produced in Easyfig and Inkscape.

## Data Availability

All data generated or analysed during this study are included in this published article [and its supplementary information files].
