# Supplementary material for: Bioinformatic prospecting and phylogenetic analysis reveals 94 undescribed circular bacteriocins and key motifs
Source: BMC Microbiol. 2020 Apr 6;20:77. doi: 10.1186/s12866-020-01772-0 (PMC7132975; doi:10.1186/s12866-020-01772-0)
Supplement: Supplementary file 2 — Additional file 2: Figure S2. Phylogenetic tree showing the two subfamilies of circular bacteriocins. Family i is shown in red, while ii is shown in blue. [file 12866_2020_1772_MOESM2_ESM.docx]

Figure S2: Phylogenetic tree showing the two subfamilies of circular bacteriocins. Family i is shown in red, while ii is shown in blue.


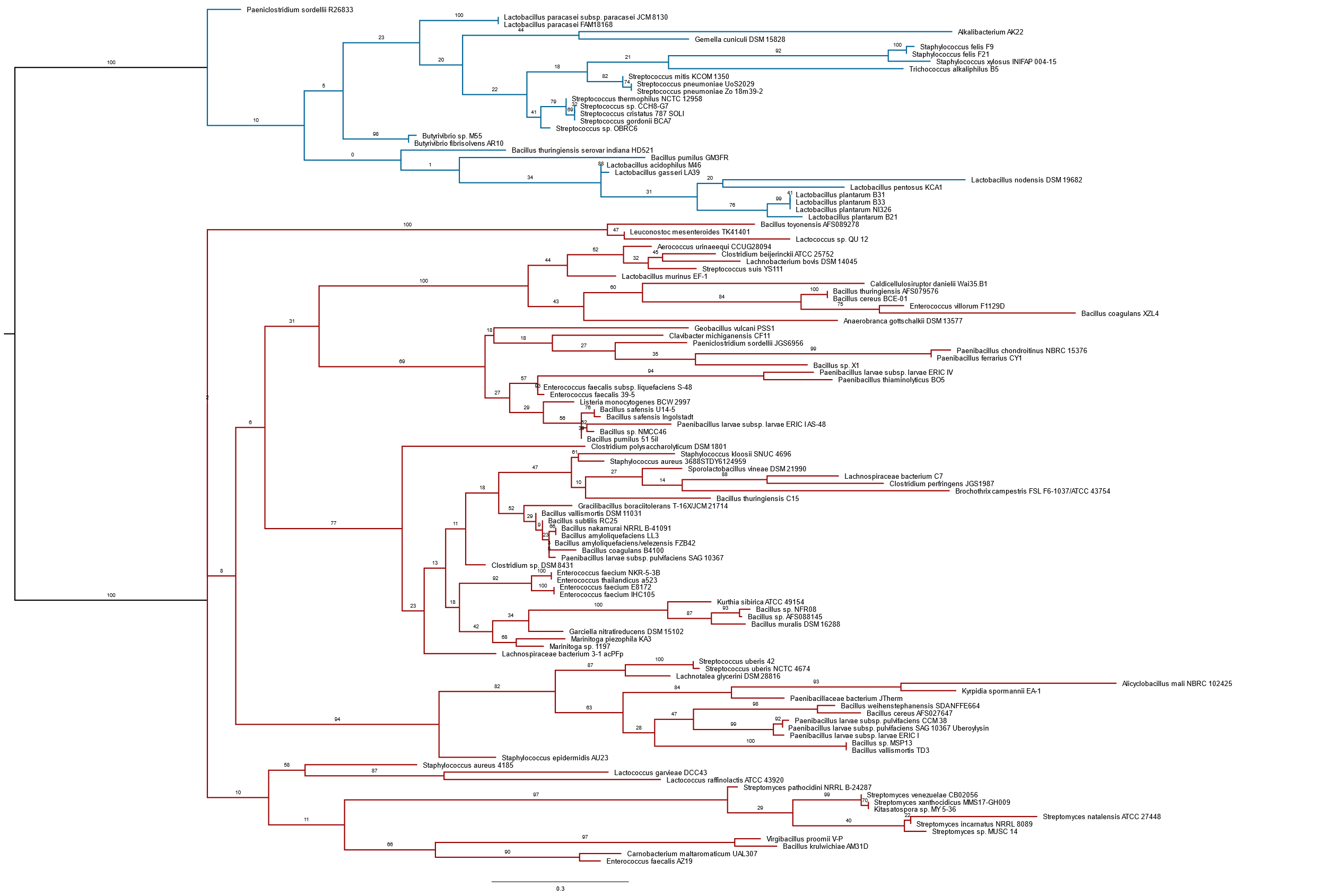


/Enterococcus faecalis strain CK135

/Bacillus pumilus B4107
