# Supplementary material for: Bioinformatic prospecting and phylogenetic analysis reveals 94 undescribed circular bacteriocins and key motifs
Source: BMC Microbiol. 2020 Apr 6;20:77. doi: 10.1186/s12866-020-01772-0 (PMC7132975; doi:10.1186/s12866-020-01772-0)
Supplement: Supplementary file 4 — Additional file 4: Figure S4. Sequence logo generated using Skylign after Clustal Omega alignment. The top logo shows family i. The bottom logo shows family ii. Family i has a sequence length of 84 due to the sequence variation within the family, resulting in a gapped alignme. [file 12866_2020_1772_MOESM4_ESM.docx]

Fig S4: Sequence logo generated using Skylign after Clustal Omega alignment. The top logo shows family i. The bottom logo shows family ii. Family i has a sequence length of 84 due to the sequence variation within the family, resulting in a gapped alignme


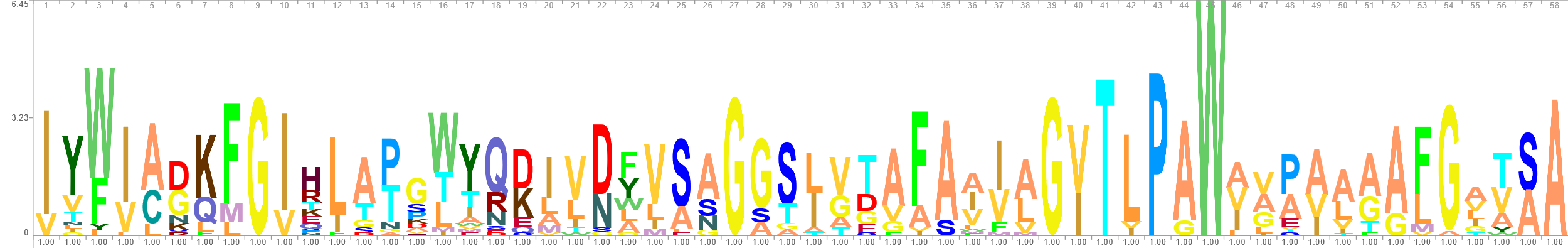

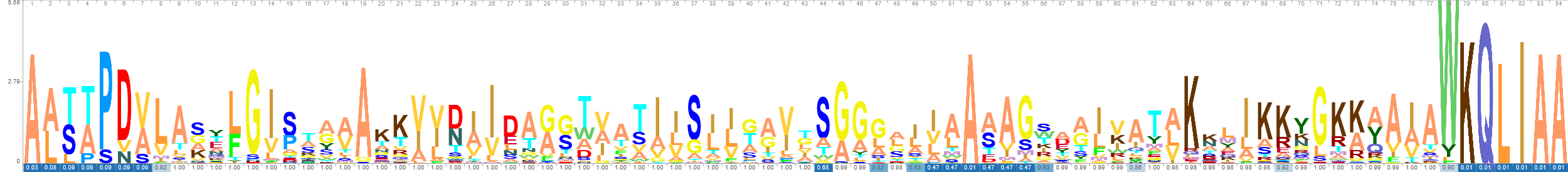


IIc i

IIc ii
