# Supplementary material for: Bioinformatic prospecting and phylogenetic analysis reveals 94 undescribed circular bacteriocins and key motifs
Source: BMC Microbiol. 2020 Apr 6;20:77. doi: 10.1186/s12866-020-01772-0 (PMC7132975; doi:10.1186/s12866-020-01772-0)
Supplement: Supplementary file 5 — Additional file 5: Figure S5. Series of figures showing the gene cluster analysis. [file 12866_2020_1772_MOESM5_ESM.docx]

Fig S5: Series of figures showing the gene cluster analysis.

Conserved gene clusters between circular bacteriocin family i


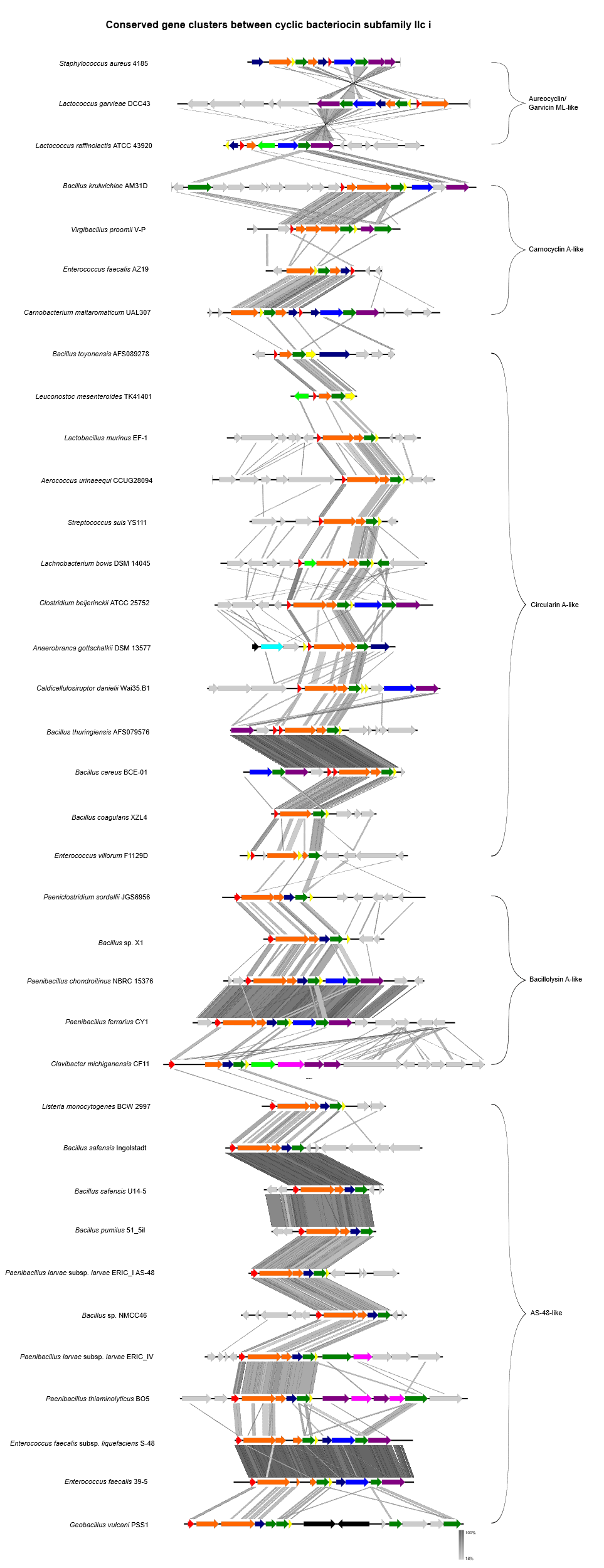


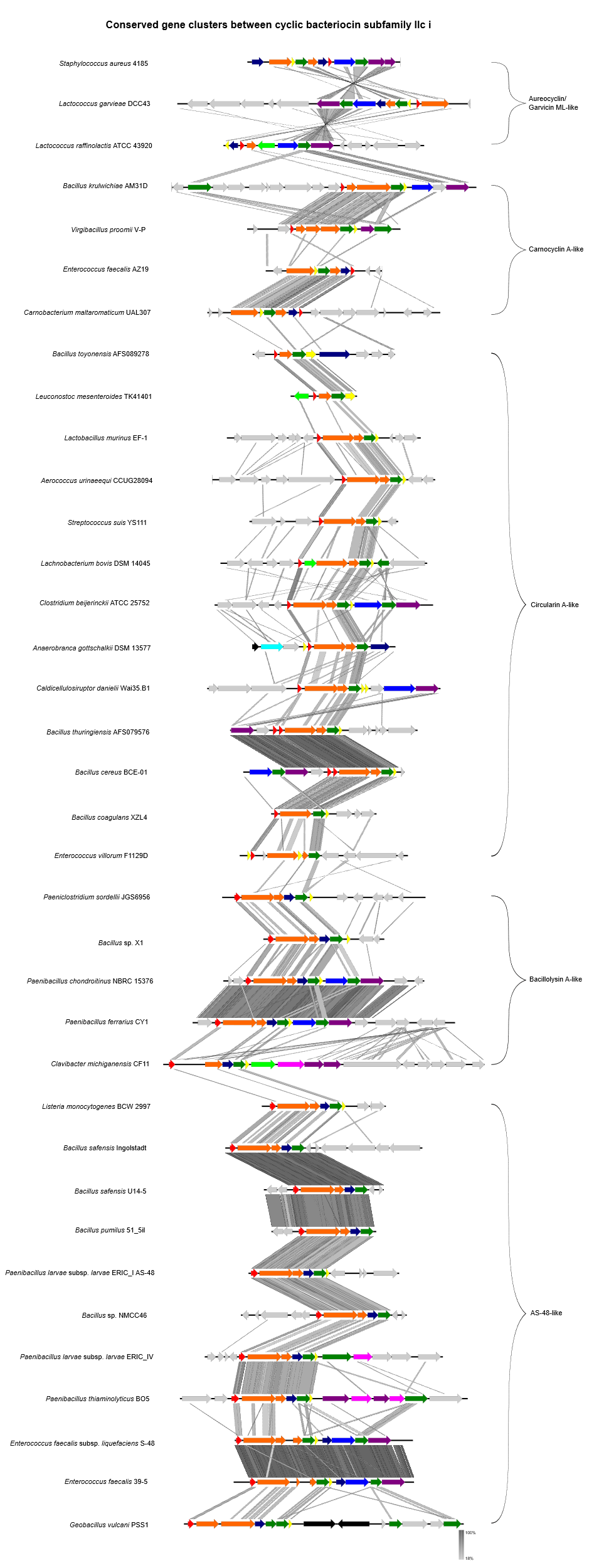


Supp figure 5a: Red shows the bacteriocin structural gene. Orange shows putative membrane proteins/stage II sporulation protein M. Yellow shows putative immunity genes such *as-48D1*. Green shows putative ABC-transporter proteins such as *as-48D* and *as-48G*. Blue shows HylD/efflux RND transporters such as as-48F. Dark blue shows other transmembrane proteins. Pink shows putative binding-protein-dependent proteins and extracellular solute-binding proteins. Purple shows ABC transporter and FtsX permeases such *as-48H*, based off the work done with AS-48 (Mercedes, Antonio et al. 2004). Light green shows transcriptional regulators. Light blue shows peptidases. Black shows phage proteins/transposons/IS elements. Brown shows Grey shows genes of unknown function which may or may not be related to circular bacteriocin production.


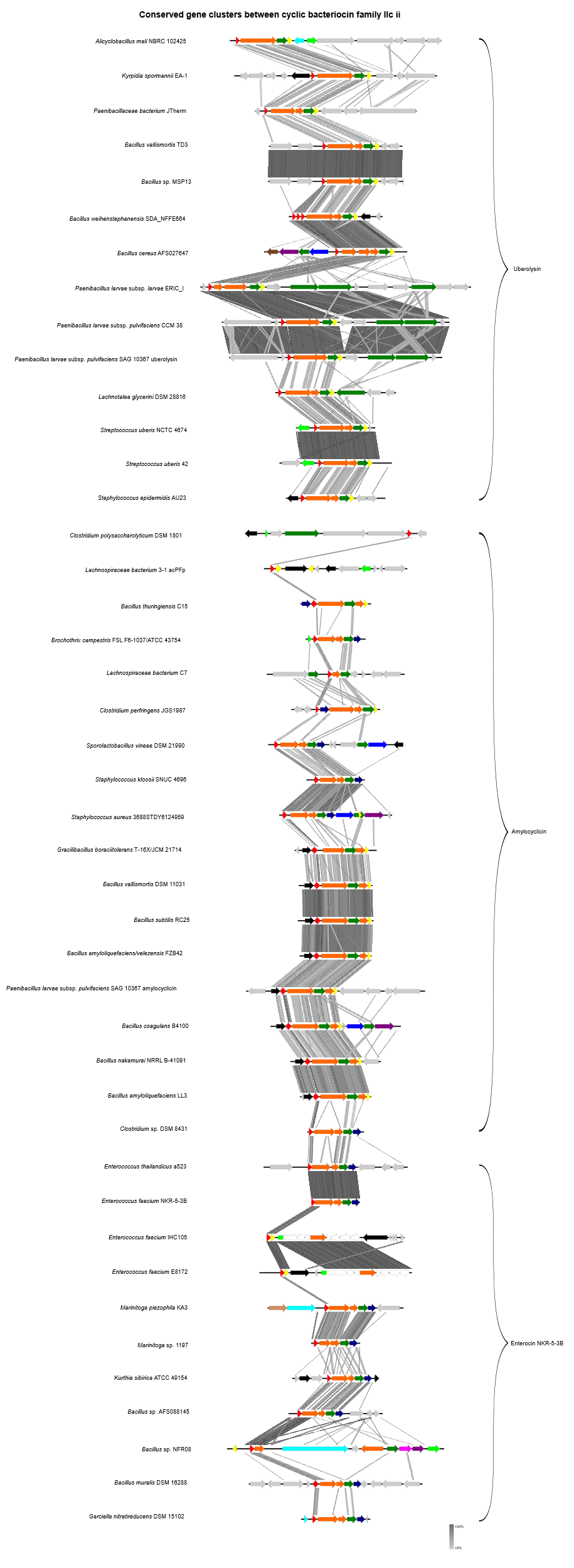


Conserved gene clusters between circular bacteriocin family i


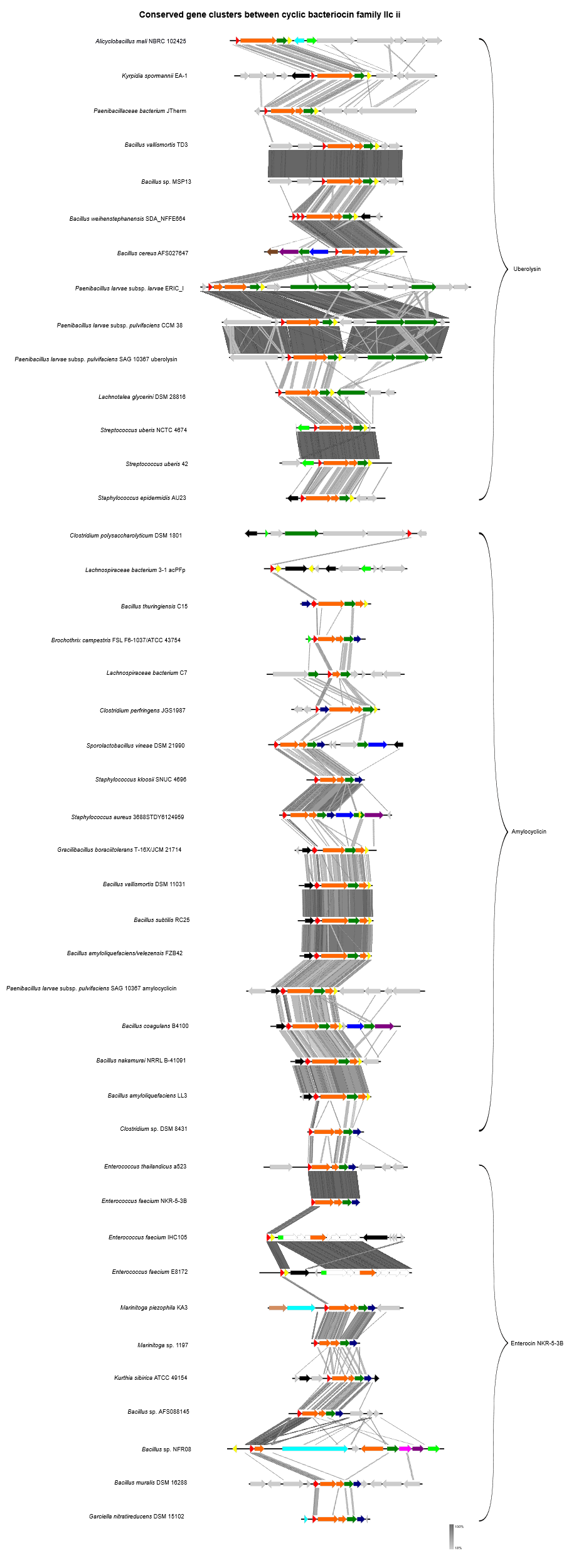


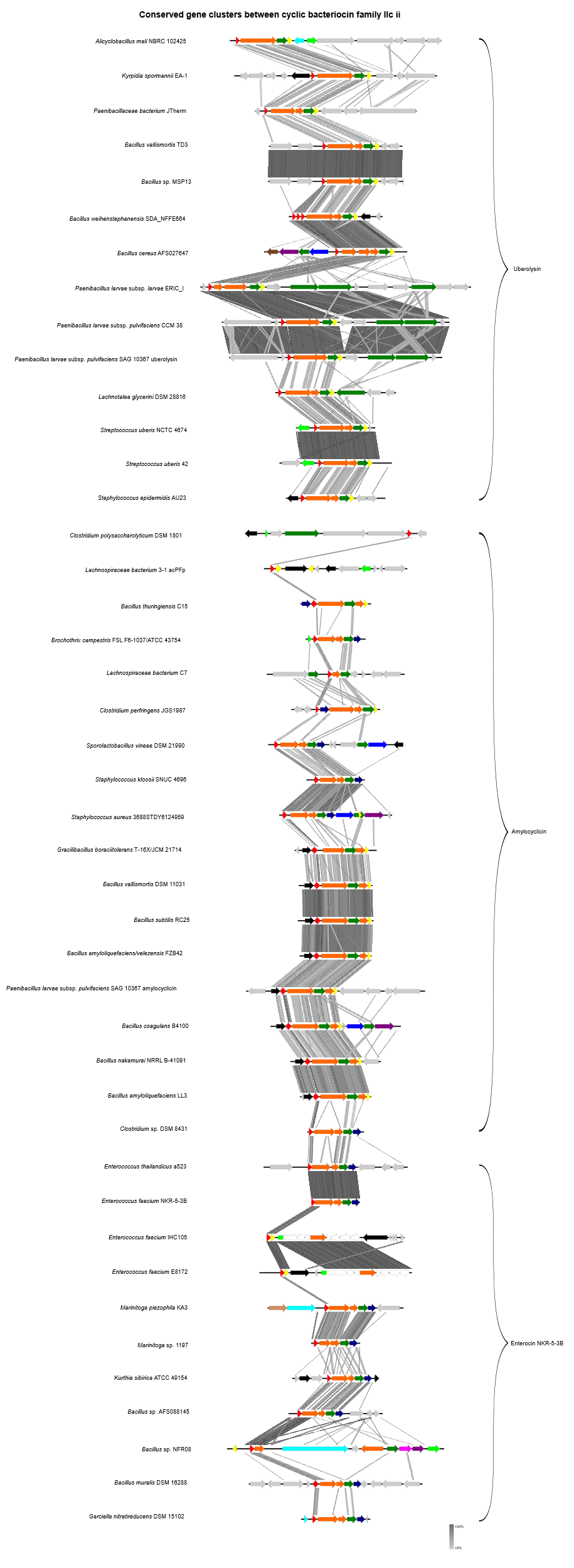


Supp figure 5b: Red shows the bacteriocin structural gene. Orange shows putative membrane proteins/stage II sporulation protein M. Yellow shows putative immunity genes such as-48D1. Green shows putative ABC-transporter proteins. Blue shows HylD/efflux RND transporters. Dark blue shows other transmembrane proteins. Purple shows ABC transporter permeases and FtsX permeases. Brown shows YIP1family proteins. Light green shows transcriptional regulators. Light blue shows peptidases. White shows genes involved with the phosphoenolpyruvate-dependent sugar phosphotransferase system. Light brown shows Major Facilitator Superfamily proteins. Pink shows putative binding-protein-dependent proteins and extracellular solute-binding proteins. Black shows mobile genetic elements such as phage proteins/transposons/IS elements. Grey shows genes of unknown function which may or may not be related to circular bacteriocin production.


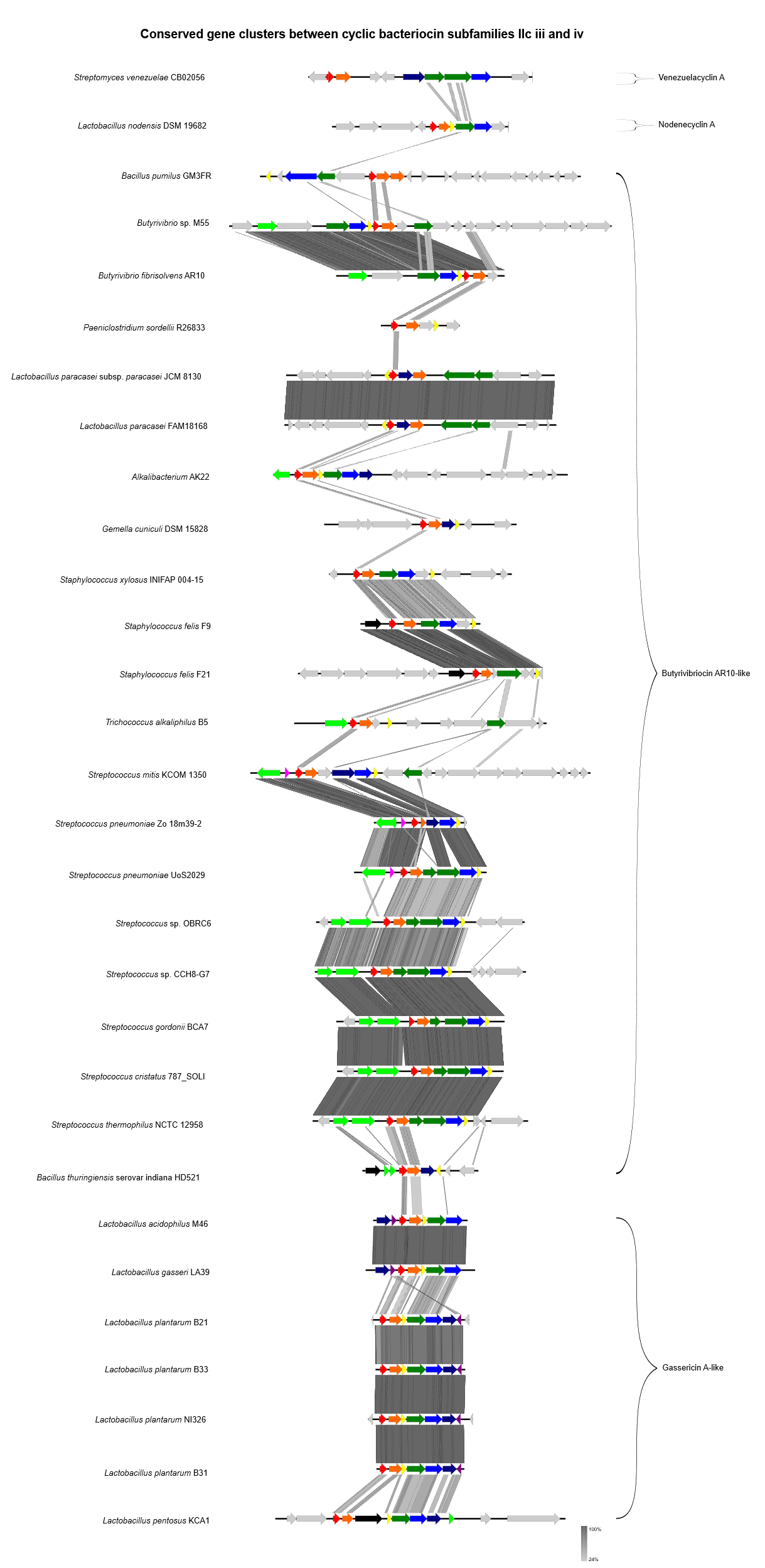


Conserved gene clusters between circular bacteriocin family ii


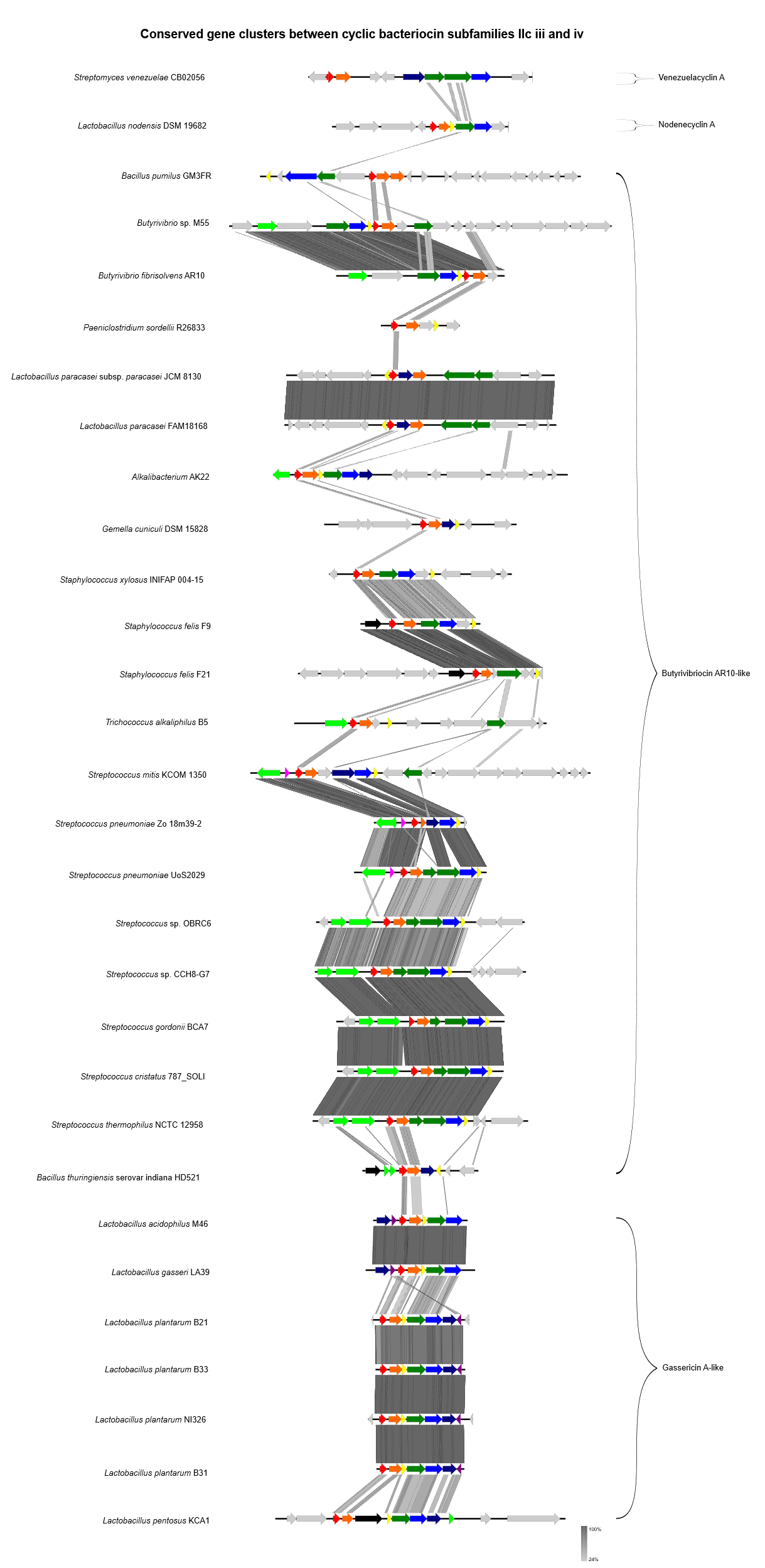


Supp figure 5c: Red shows the putative cyclic bacteriocin structural genes. Orange shows putative membrane proteins/stage II sporulation proteins. Yellow shows putative immunity proteins. Green shows putative ABC-transporter proteins. Blue shows a putative ABC-II transporter permease. Dark blue shows another putative transmembrane protein. Purple shows a gene of unknown function, possibly involved in immunity. Light green shows XRE transcriptional/response regulators. Pink shows putative secreted protein. Black shows phage proteins/transposons/IS elements. Grey shows genes of unknown function which may or may not be related to circular bacteriocin production.


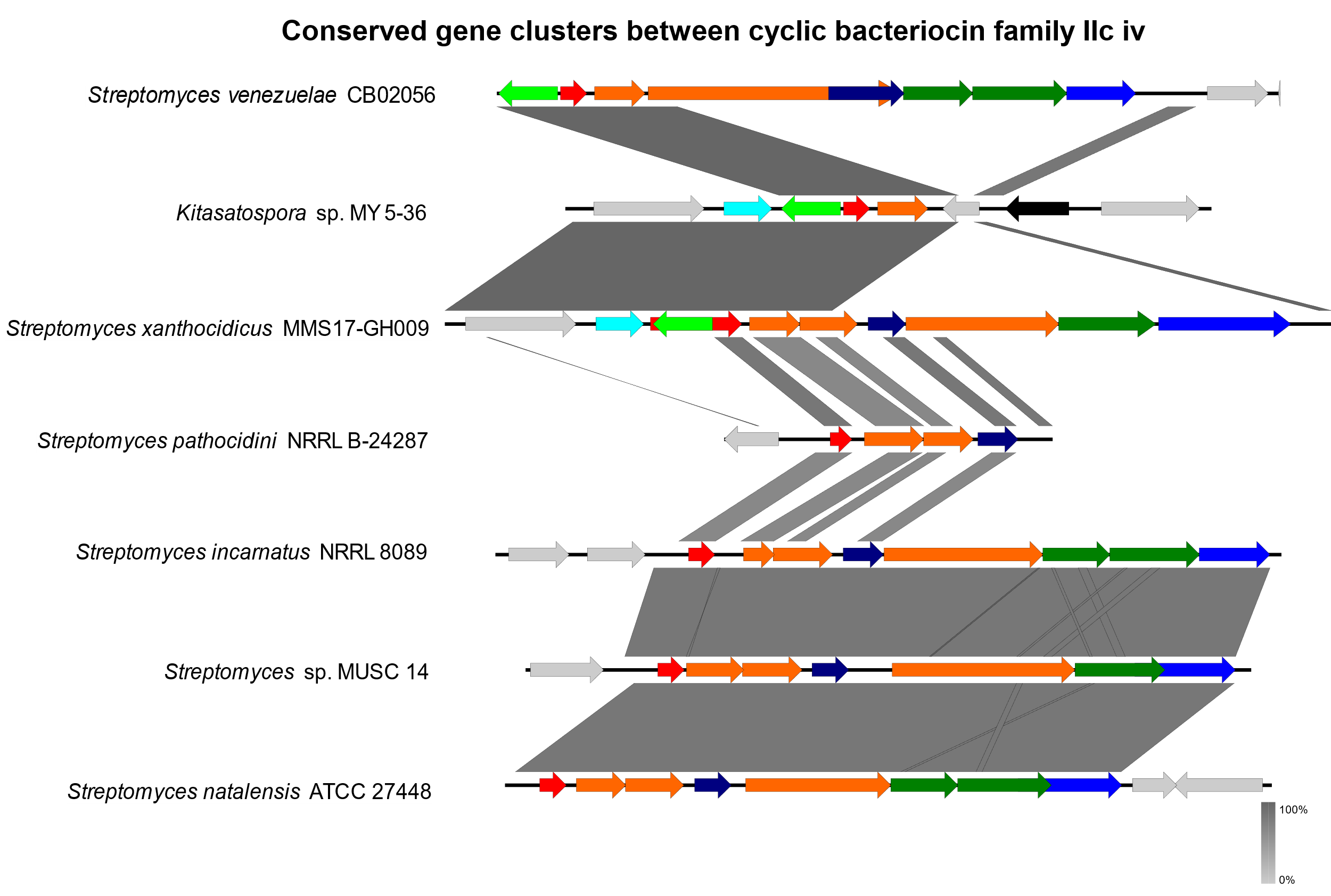


Supp figure 5d: Red shows the putative cyclic bacteriocin structural genes. Orange shows putative membrane proteins/stage II sporulation proteins. Yellow shows putative immunity proteins. Green shows putative ABC-transporter proteins. Blue shows a putative ABC-II transporter permease. Dark blue shows another putative transmembrane protein. Light green shows XRE transcriptional/response regulators. Light blue shows peptidases. Black shows phage proteins/transposons/IS elements. Grey shows genes of unknown function which may or may not be related to cyclic bacteriocin production.

Conserved gene clusters between circular bacteriocin family i
