# Supplementary material for: Bioinformatic prospecting and phylogenetic analysis reveals 94 undescribed circular bacteriocins and key motifs
Source: BMC Microbiol. 2020 Apr 6;20:77. doi: 10.1186/s12866-020-01772-0 (PMC7132975; doi:10.1186/s12866-020-01772-0)
Supplement: Supplementary file 6 — Additional file 6: Table S1. Summary of circular bacteriocin gene clusters and their general distribution throughout the circular bacteriocin subfamilies. Not every circular bacteriocin cluster within each subfamily was predicted to contain each of the genes here, though generally this was the case. [file 12866_2020_1772_MOESM6_ESM.docx]

Table S1: Summary of circular bacteriocin gene clusters and their general distribution throughout the circular bacteriocin subfamilies. Not every circular bacteriocin cluster within each subfamily was predicted to contain each of the genes here, though generally this was the case.

| **Gene** | **Predicted role** | **Subfamilies absent from** |
| --- | --- | --- |
| Structural circular bacteriocin | Bacteriocin | None |
| Putative transmembrane protein/SpoIIM | Transmembrane protein, possibly part of multicomponent ABC transporter | None |
| ABC transporter | Cleave signal peptide and export bacteriocin to periplasm/extracellular space | None |
| ABC-II/FtsX permease | Possibly transport immunity protein | Enterocin-NKR-3B, amylocyclicin, uberolysin, carnocyclin |
| Immunity protein | Immunity against bacteriocin | None |
| HylD/efflux RND transporters | Possibly involved in transport of bacteriocin from periplasm to extracellular space or additional immunity | Almost all, present only in few individual clusters within subfamilies |
