# Supplementary material for: Bioinformatic prospecting and phylogenetic analysis reveals 94 undescribed circular bacteriocins and key motifs
Source: BMC Microbiol. 2020 Apr 6;20:77. doi: 10.1186/s12866-020-01772-0 (PMC7132975; doi:10.1186/s12866-020-01772-0)
Supplement: Supplementary file 7 — Additional file 7: Figure S6. Bacillocyclin subfamily cluster analysis. Diagram showing the conserved genes within the putative bacillocyclin subfamily. Arrows show putative genes within the gene cluster. Red shows the bacteriocin structural gene. Orange shows putative membrane proteins/stage II sporulation protein M. Yellow shows putative immunity genes such as-48D1. Green shows putative ABC-transporter proteins such as as-48D and as-48G. Blue shows HylD/efflux RND transporters such as as-48F. Dark blue shows other transmembrane proteins. Pink shows putative binding-protein-dependent proteins and extracellular solute-binding proteins. Purple shows ABC-II/FtsX permeases such as-48H, based off the work done with AS-48 [4]. Grey shows genes of unknown function which may or may not be related to circular bacteriocin production. Figure produced in Easyfig and Inkscape. [file 12866_2020_1772_MOESM7_ESM.docx]

Fig S6: Bacillocyclin subfamily cluster analysis


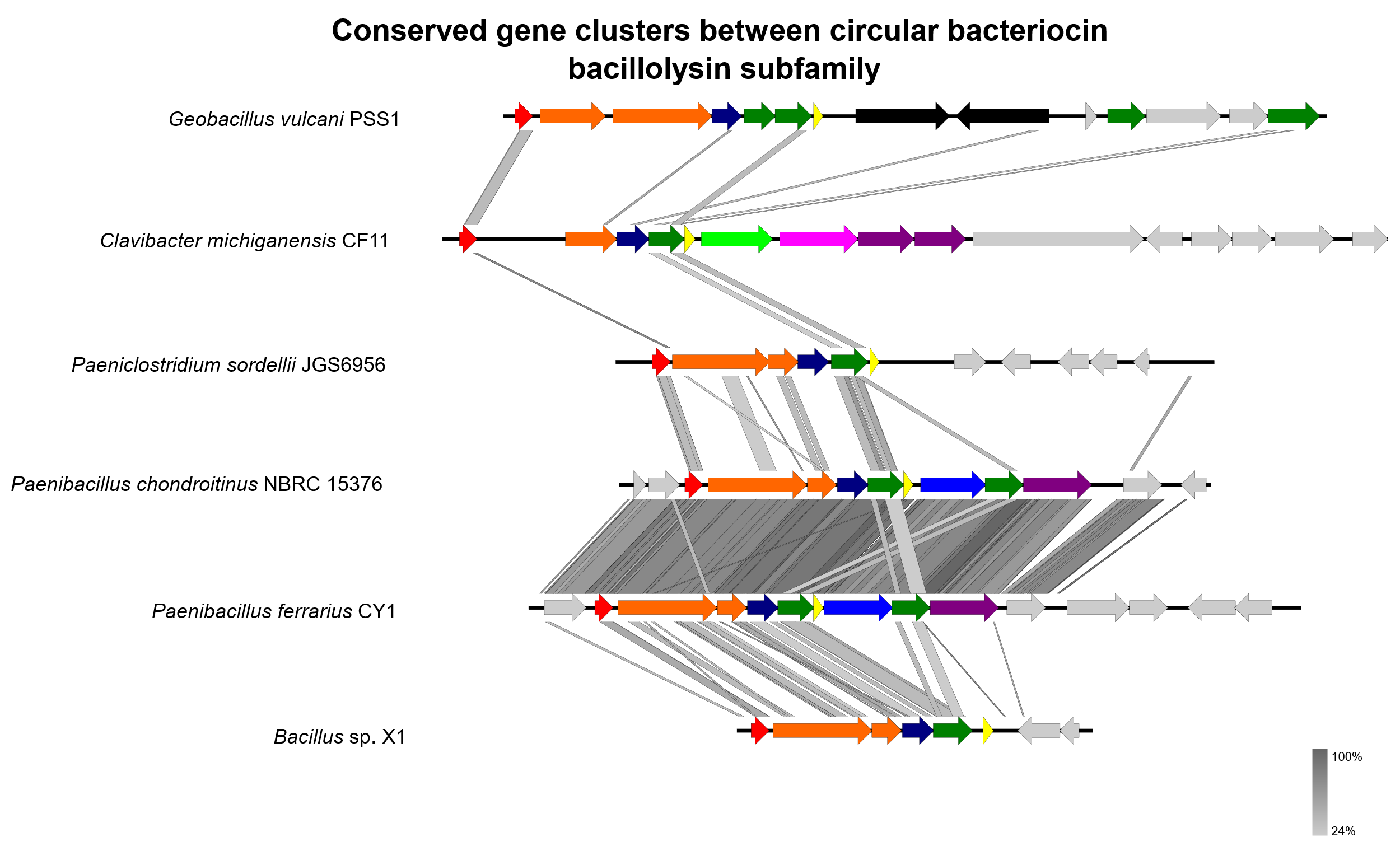


Diagram showing the conserved genes within the putative bacillocyclin subfamily. Arrows show putative genes within the gene cluster. Red shows the bacteriocin structural gene. Orange shows putative membrane proteins/stage II sporulation protein M. Yellow shows putative immunity genes such *as-48D1*. Green shows putative ABC-transporter proteins such as *as-48D* and *as-48G*. Blue shows HylD/efflux RND transporters such as as-48F. Dark blue shows other transmembrane proteins. Pink shows putative binding-protein-dependent proteins and extracellular solute-binding proteins. Purple shows ABC-II/FtsX permeases such *as-48H*, based off the work done with AS-48 [4]. Grey shows genes of unknown function which may or may not be related to circular bacteriocin production. Figure produced in Easyfig and Inkscape.

**Conserved putative circular bacteriocin gene clusters within the bacillocyclin subfamily**
